# Cardiovascular Manifestations and Outcomes in Patients with Scrub Typhus Admitted to a Tertiary Care Center in the Coastal Karnataka Region in India

**DOI:** 10.3390/diseases13080270

**Published:** 2025-08-20

**Authors:** Mugula Sudhakar Rao, Jyothi Samanth, Swathi Poojary, Krishnananda Nayak, Shubha Srinivas, Thrupthi Naik

**Affiliations:** 1 Department of Cardiology, Manipal Hospital, Bangalore 560041, India; 2 Department of Cardiovascular Technology, Manipal College of Health Professions, Manipal Academy of Higher Education, Manipal 576104, India

**Keywords:** scrub typhus, myocarditis, heart failure, LV dysfunction

## Abstract

**Introduction**: Scrub typhus is a mite-borne infectious disease caused by “*Orientia tsutsugamushi*”, a bacterium that was formerly classified under the genus Rickettsia. It is transmitted to humans through the bites of infected chigger mites (larval trombiculid mites). However, clinical data on the cardiac manifestations of scrub typhus and their outcomes remain limited. **Methods**: This research was retrospectively conducted at a tertiary care hospital in South India. The study included all patients admitted from January 2016 to September 2021 who fulfilled the clinical criteria for a diagnosis of scrub typhus. Data were collected for 426 patients. Patients with previously diagnosed heart disease and mixed infections (leptospirosis, dengue fever, blood culture positivity, and COVID-19 positivity) were excluded. Comprehensive assessments of clinical presentation, electrocardiography (ECG), 2D echocardiography, and outcomes, including all-cause mortality and probable myocarditis, were performed. Multivariate regression analysis was performed to identify independent predictors of all-cause mortality and probable myocarditis. **Results**: Out of 426 patients, 200 (46.9%) were male and 226 (53.1%) were female. The mean age at presentation was 49.29 ± 14.43 years. A total of 108 (25.4%) patients had diabetes and 82 (19.25%) had hypertension. Sinus tachycardia (29.3%) was the most frequent ECG finding. Echocardiographic evidence of probable myocarditis was observed in 20 (4.7%) patients, while 6 (1.4%) patients had isolated RV dysfunction, 4 (0.9%) had biventricular dysfunction, 7 (1.6%) had significant pulmonary hypertension, and 40 (9.4%) had trivial pericardial effusion. A total of 78 (18.3%) patients had acute respiratory distress syndrome. All-cause mortality was observed in 12 (2.8%) patients. A total of 56 (13.1%) patients developed multiorgan dysfunction syndrome (MODS) during their hospitalization. A total of 78 (18.3%) patients were documented to have acute kidney injury (AKI), and 22 (5.2%) patients underwent hemodialysis. Multivariable binary logistic regression analysis revealed that probable myocarditis and MODS were independent predictors of mortality among patients with scrub typhus, and age, female gender, and LV systolic dysfunction were identified as predictors of overall complications, including mortality, probable myocarditis, congestive heart failure, MODS, AKI, and the need for hemodialysis. **Conclusions**: Probable myocarditis was the most frequent cardiac manifestation noted in patients with scrub typhus, and in addition to MODS, probable myocarditis was an independent predictor of mortality in this cohort. Thus, it is crucial to maintain clinical vigilance regarding the cardiac status of such patients.

## 1. Introduction

Scrub typhus is an acute febrile illness caused by “*Orientia tsutsugamushi*”, a gram-negative intracellular bacterium transmitted through the bites of infected chigger mites. “*Orientia tsutsugamushi*” was previously grouped under the genus Rickettsia, and scrub typhus is still often categorized among the rickettsial diseases due to overlapping clinical features and pathogenesis. This disease is prevalent within the geographical region known as the “tsutsugamushi triangle” [1,2,3]. Untreated scrub typhus has a reported mortality rate of up to 6.0%, whereas appropriate antibiotic therapy reduces the mortality rate to approximately 1.4% [4]. Transmission occurs through the larval stage of trombiculid mites (chiggers), which is the only parasitic stage to humans and animals. These chiggers commonly infest wild rodents, particularly those of the Rattus genus, and humans become incidental hosts when exposed to mite-infested habitats [5]. The most common cardiovascular manifestation of scrub typhus is myocarditis, which manifests as cellular infiltration involving layers of the heart, mainly the inner and outer layers. Sinus arrhythmia, bradycardia, prominent U waves, and T wave abnormalities are common electrocardiographic findings in scrub typhus patients [2,6]. Regional and global left ventricular (LV) dysfunction can occur along with LV diastolic dysfunction. Other less common features are pericardial effusion, chamber dilatation, and increased chamber thickness [7]. Frequent cardiac manifestations have been observed in scrub typhus patients, but they are associated with increased morbidity and not mortality [2]. There is limited evidence on cardiovascular outcomes and their predictors among scrub typhus patients. This retrospective study was performed to assess cardiovascular manifestations and outcomes, including their predictors, among patients with scrub typhus admitted to a tertiary care center in coastal Karnataka.

## 2. Materials and Methods

**Figure 1:**
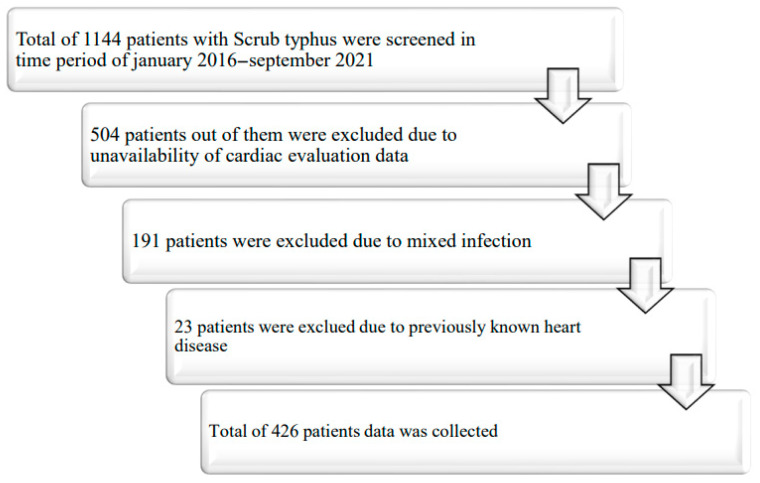
Flowchart of patient inclusion and exclusion criteria.

This retrospective study was conducted at a tertiary care hospital in South India. All patients admitted between January 2016 and September 2021 who met the clinical criteria for scrub typhus were included in the study (Figure 1). Institutional ethics committee approval was obtained before the study. Patients who were previously diagnosed with heart disease (ischemic heart disease, valvular heart disease, congenital heart disease, cardiomyopathies, or pre-existing arrhythmias) or had mixed infections (leptospirosis, dengue, blood culture positive, or COVID-19 positive) were excluded since patients with this infection may also have the same cardiac abnormalities. Patients were also excluded if complete cardiac evaluation data, including electrocardiography and echocardiography data, were unavailable.

Baseline characteristics, including age, gender, and comorbidities, including diabetes and hypertension history, were collected. Baseline data, including cardiac troponin-T, CK-MB, NT-proBNP, C-reactive protein, complete blood counts, renal parameters, liver function tests, and procalcitonin, were collected when available.

Baseline electrocardiogram findings at the time of admission revealed heart rate, conduction disturbance, rhythm disturbance, ST-T changes, QTc interval, and repolarization abnormalities. Rhythm abnormalities such as atrial fibrillation (AF) and ventricular tachycardia (VT) were also noted if they occurred during hospitalization. Heart rate less than 60 bpm was defined as sinus bradycardia, and heart rate > 100 bpm was defined as sinus tachycardia. A QTc interval > 460 ms in males and > 480 ms in females was considered a prolonged QTc interval.

Two-dimensional echocardiography data were collected to assess the end-systolic dimension (ESD), end-diastolic dimension (EDD), end-systolic and end-diastolic volumes (ESV and EDV), ejection fraction (EF), fractional shortening (FS), regional wall motion abnormalities (RWMA), global hypokinesia, regurgitation of mitral and tricuspid valves, right ventricular systolic pressure (RVSP), pericardial effusion, and both isolated LV dysfunction and isolated right ventricular (RV) dysfunction, as well as biventricular dysfunction.

Right ventricular systolic pressure ≥ 50 mmHg was considered to indicate significant pulmonary artery hypertension.

Probable myocarditis was defined as

(1) Signs and symptoms of congestive heart failure (CHF) with new onset of left/right ventricular dysfunction in patients with previously normal echocardiographic findings on admission;
(2) Signs and symptoms of heart failure and left ventricular dysfunction with evidence of myocardial necrosis (troponin T > 0.1).

Isolated left ventricular dysfunction was considered for patients with an EF < 50%, irrespective of elevated troponin T. Isolated right ventricular dysfunction was considered when there was evidence of right atrium/right ventricular dilatation, low-pressure tricuspid regurgitation, and/or tricuspid annular planar systolic excursion (TAPSE) < 16 mm. Significant regurgitation of valves was considered when more than mild MR and TR were present. Pericardial effusion was diagnosed as an echo-free space between the two layers of the pericardium, and >10 mm of effusion was considered significant.

The primary outcomes were all-cause in-hospital mortality and probable myocarditis. Other outcome measures were noted, such as the need for hemodialysis, hospitalization days, multiorgan dysfunction syndrome (MODS), and resuscitated cardiac arrest.

IBM SPSS Statistics 22 software was used for data analysis. The proportions, mean values, and standard deviations for normally distributed data and medians (interquartile ranges) for skewed data were reported. To determine associations between 2 categorical variables, a chi-square test was applied. To identify the independent predictors of outcomes such as probable myocarditis and mortality, a multivariable regression analysis was performed. A *p* value of <0.05 is considered statistically significant.

## 3. Results

A total of 1144 patients diagnosed with scrub typhus were admitted between January 2016 and September 2021. Of these, 718 patients were excluded for the following reasons: 504 patients did not undergo a complete cardiac evaluation during the study period; 191 patients had mixed infections; and 23 patients had a pre-existing diagnosis of heart disease. Hence, out of 1144 patients, 712 were excluded from this study, and data from 426 patients were acquired (Figure 1). Of the 426 patients, 200 (46.9%) were male and 226 (53.1%) were female. The mean age of the patients at presentation was 49.29 ± 14.44 years. The mean ages of the males and females were 50.24 ± 15.2 years and 48.46 ± 13.69 years, respectively. A total of 108 (25.4%) patients had a history of diabetes mellitus, 82 (19.25%) patients had hypertension, and 78 (18.3%) had acute respiratory distress syndrome (Table 1). The clinical course during admission to the hospital revealed that the median duration of hospital stay was 8 (IQR 6, 8) days, and 132 (31%) patients had longer hospital stays (≥ 10 days). Troponin T and NT-proBNP were available for only 190 and 112 patients, respectively. Eleven patients had CK-MB, 182 patients had CRP, and 148 patients had available procalcitonin data. The median values and interquartile ranges are reported in Table 1.

### 3.1. Electrocardiographic Data and Echocardiographic Data

Most of the patients had sinus rhythm (59.9%), and the most common abnormal ECG finding was sinus tachycardia (29.3%). Other ECG findings included ST-T changes (7.5%), bradycardia (2.8%), atrial fibrillation (2.1%), left bundle branch block (0.5%), right bundle branch block (0.2%), and myocardial infarction (0.2%) (Table 1).

Most of the patients had a normal LV ejection fraction. Probable myocarditis was observed in 20 (4.7%) patients. Global LV dysfunction was observed in 20 (4.7%) patients, and regional wall motion abnormalities were noted in 3 (0.7%) patients. Six (1.4%) patients had isolated RV dysfunction. Seven (1.6%) patients had significant pulmonary hypertension, and four (0.9%) had biventricular dysfunction. Pericardial effusion was observed in 40 (9.4%) patients, 34 (8%) of whom had only trivial pericardial effusion. Seven (1.6%) patients had mild pericardial effusion, and one (0.2%) patient had moderate pericardial effusion. Moderate MR was noted in 29 (6.8%) patients, and severe MR was noted in 4 (0.9%) patients. Significant tricuspid valve abnormalities were detected in 55 (12.9%) patients, and moderate aortic regurgitation was detected in 2 (0.5%) patients. Diastolic dysfunction was Grade I in 114 (26.8%) patients, Grade II in 1 (0.2%) patient, and Grade III in 1 (0.2%) patient (Table 1).

### 3.2. Outcome Measures

**Figure 2:**
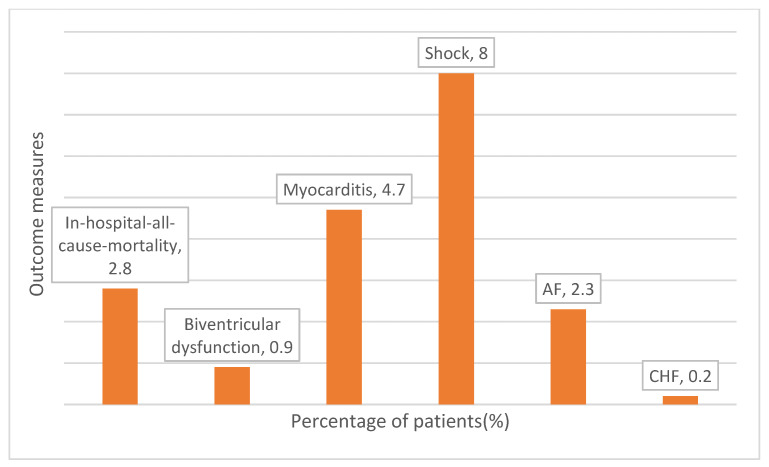
Patient distribution percentages according to outcome measures.

Twelve (2.8%) patients died in hospital. Probable myocarditis was noted in 20 (4.7%) patients, atrial fibrillation in 9 (2.1%), and congestive cardiac failure in 1 (0.2%) patient (Table 2 and Figure 2). A total of 56 (13.1%) patients developed MODS during their hospitalization. A total of 78 (18.3%) patients were documented to have acute kidney injury (AKI), and 22 (5.2%) patients underwent hemodialysis (Table 2). Multivariable binary logistic regression analysis that the presence of probable myocarditis and MODS were independent predictors of mortality among patients with scrub typhus (Table 3). Additionally, age, female gender, and LV dysfunction were found to be independent predictors of overall complications (mortality, probable myocarditis, MODS, CHF, AKI, and need for hemodialysis).

## 4. Discussion

Scrub typhus is an acute febrile illness transmitted from bites of mite larvae (chigger) bacteria. Here, humans can be accidental hosts. These mites are usually found in agricultural fields and sandy areas of Asia, mainly in the southern and eastern zones of India, Korea, Sri Lanka, Thailand, Japan, and Pakistan called the Tsutugamushi Triangle. Scrub typhus mainly affects the lungs, heart, liver, spleen, and central nervous system. Cardiac involvement in scrub typhus has significant implications for mortality rates and other health outcomes. Among the cardiac manifestations, sinus tachycardia is the most frequent electrocardiogram (ECG) finding and is accompanied by other nonspecific ECG anomalies. Important echocardiographic abnormalities include probable myocarditis, systolic and diastolic dysfunction of the left ventricle, regional abnormalities in wall motion, and pericardial effusion.

The central objective of our research was to comprehensively explore the diverse cardiac manifestations associated with scrub typhus and identify potential prognostic indicators among patients with this condition. In our research, the average age of the participants was 49.3 ± 14.4 years. Of the participants 46.9% (200) were males, while 53.1% (226) were females. This distribution of gender exhibited a slight female predominance, aligning with similar patterns observed in other studies [8,9].

Regarding cardiac rhythm, most patients (59.9%) in our study exhibited sinus rhythm, with the most frequently encountered ECG abnormality being sinus tachycardia (29.3%). Additional ECG anomalies were identified, including ST-T changes (7.5%), sinus bradycardia (2.8%), atrial fibrillation (2.1%), left bundle branch block (LBBB) (0.5%), right bundle branch block (RBBB) (0.2%), and myocardial infarction (0.2%), all of which are classified as non-specific findings. Similarly, a study by George Watt et al. in Northern Thailand involving 29 patients showed that 22 had a normal ECG, while the remaining 7 displayed ST-T changes, U waves, and premature ventricular beats [10]. Dong-Hyun Choi et al.’s study identified ECG abnormalities in 43.9% of patients, with 18.8% experiencing arrhythmias, 15.1% indicating signs of ischemia, and 19.4% exhibiting QT prolongation [11]. Another study by Rajapakse S et al. revealed that approximately half of patients exhibited ECG irregularities, including bradycardia and other deviations such as sinus tachycardia, QT prolongation, atrial flutter or fibrillation, ventricular premature beats, heart block, prominent U waves, and ST-T changes [12]. It is still uncertain how ECG changes occur in patients with scrub typhus [11].

In our investigation, probable myocarditis was identified in 4.7% of the patients. Our study also revealed that 26.8% of patients experienced mild left ventricular (LV) diastolic dysfunction, while only 0.7% showed regional wall motion abnormalities (RWMAs). Karthik Gunashekaran et al. reported that among 81 patients, 17 individuals (21%) exhibited probable myocarditis and mild LV diastolic dysfunction was observed in 18 patients (22.2%), with 14.8% demonstrating RWMAs and 51% indicating pericardial effusion [2]. However, our study reported a lower prevalence of pericardial effusion, at 9.4%, of which 8% exhibited trivial pericardial effusion, 1.6% exhibited mild pericardial effusion, and 0.2% exhibited moderate pericardial effusion, aligning with the findings of Ki Woon et al., whose study showed a 6.7% occurrence of pericardial effusion [12]. Additionally, our study revealed other echocardiographic characteristics, including globally hypokinetic LV (4.7%), right ventricular (RV) dysfunction (1.4%), biventricular failure (0.9%), and notable pulmonary hypertension (1.6%).

Previous research by Ki Woon Kang et al. revealed that bilirubin and paroxysmal atrial fibrillation were predictors of acute myocarditis [12]. Moreover, Dong-Hyun Choi et al. established that patients with prolonged QT intervals had severe scrub typhus [11]. An investigation by Rajapakse S et al. highlighted the significant role of eschar in suspected scrub typhus [13]. Furthermore, our study indicated that probable myocarditis and MODS were independent predictors of mortality (adjusted odds ratio: 9.79 [95% CI: 1.88–50.79] and 7.45 [95% CI: 1.65–33.66], respectively; *p* < 0.05), with mortality rates reaching 2.8% among patients with scrub typhus. It is generally believed that myocarditis is a rare but severe complication of scrub typhus that can lead to high mortality rates and may present itself later [9,14,15]. The study also demonstrated that age, female, and LV systolic dysfunction were predictors of overall complications, including mortality, probable myocarditis, CHF, MODS, AKI, and the need for hemodialysis. While Karthik Gunashekaran et al.’s study suggested the presence of myocarditis or myocardial dysfunction in patients, the ultimate implications of this issue remain uncertain [2]. It is important to note that detecting probable myocarditis early through echocardiography can significantly reduce the mortality rate of patients with scrub typhus.

## 5. Limitations

First, this was a retrospective study in which 718 patients were excluded (504 patients were excluded because they did not undergo cardiac evaluation during admission and 191 patients were excluded due to mixed infection), and 23 patients were already diagnosed with heart disease. These exclusions may introduce selection bias and affect the generalizability of the study. Second, this study revealed a gender distribution with a notable predominance of females. Achieving a more balanced gender representation would enhance this study’s comprehensiveness. Third, this study did not account for medications used in the prevention of cardiovascular diseases. The absence of data on medications such as statins, antihypertensive drugs, and antiplatelet drugs represents a limitation. The exclusion of these studies could limit the ability to determine cardiovascular disease prevention strategies. Fourth, patient data were selected based on International Classification of Diseases (ICD) codes. This method of data selection carries the potential for misclassification bias. Additionally, cardiac biomarkers such as troponin and NT-proBNP were not available for all patients, limiting the interpretation of myocardial injury. Furthermore, the diagnosis of myocarditis in our study was not confirmed by cardiac magnetic resonance imaging or endomyocardial biopsy, which are considered gold-standard modalities. Given the inconsistent availability of cardiac biomarkers and the lack of advanced diagnostic tools, the diagnosis should be interpreted as probable myocarditis, and we acknowledge this as a major diagnostic limitation.

## 6. Conclusions

Probable myocarditis was the most frequent cardiac manifestation noted in patients with scrub typhus, and in addition to MODS, probable myocarditis was an independent predictor of mortality in patients with scrub typhus. Notably, age, female gender, and LV systolic dysfunction appeared to influence outcomes related to overall complications (mortality, probable myocarditis, MODS, CHF, AKI, and the need for hemodialysis). This information highlights the crucial need for healthcare practitioners to identify the prevailing cardiovascular risks in patients and consider appropriate cardiac monitoring when there are suspicions based on clinical presentation.

## Figures and Tables

**Table 1:** Baseline characteristics.

Parameters	Values
Mean age (years, mean ± SD)	49 ± 14
Gender	
Male n (%)	200 (46.9)
Female n (%)	226 (53.1)
**Comorbidities**	
Diabetic n (%)	108 (25.4)
Hypertensive n (%)	82 (19.2)
ARDS n (%)	78 (18.3)
**Laboratory findings Median (Q1, Q3)**	
Cardiac Trop T, [Normal range: up to 0.02 ng/mL]	0.0120 (0.004–0.031)
CK-MB, [Normal range: 5–25 IU/L]	92.5 (58.25–141.75)
NT-proBNP, [Normal range: 40–125 pg/mL]	1091.5 (236.5–3933.3)
CRP, [Normal range: 0–6 mg/L]	120.5 (64–192.4)
Haemoglobin, Normal range: 13.0–17.0 g/dL]	11.6 (10.1–12.7)
Serum creatinine, [Normal range: 0.7–1.2 mg/dL]	0.92 (0.7–1.34)
WBC count, [Normal range: 4.0–10.0 × 10^3^/µL]	9 (6.4–12.4)
Platelet count, [Normal range: 150–400 × 10^3^/µL]	101 (54–165)
Total bilirubin, [Normal range: 0.3–1.2 mg/dL]	1.4 (0.7–3.9)
Direct bilirubin, [Normal range: 0.0–0.4 mg/dL]	0.9 (0.3–3.4)
AST, [Normal range: up to 40 IU/L]	106 (64–164)
ALT, [Normal range: up to 41 IU/L]	76.5 (50–120)
Procalcitonin, [Normal range: <0.1 ng/mL]	2.15 (1.1–4.3)
**Electrocardiographic parameters**	
Sinus rhythm n (%)	255 (59.9)
Tachycardia n (%)	125 (29.3)
Bradycardia n (%)	12 (2.8)
LBBB n (%)	2 (0.5)
RBBB n (%)	1 (0.2)
ST-T changes n (%)	32 (7.5)
Atrial fibrillation n (%)	9 (2.1)
Myocardial infarction n (%)	1 (0.2)
**Echocardiography Findings**	
EDD (Mean ± SD)	45 ± 5
ESD (Mean ± SD)	28.8 ± 4.5
EF (Mean ± SD)	63.9 ± 5.9
FS (Mean ± SD)	34.4 ± 4.4
RVSP (Mean ± SD)	29 ± 7.9
RVSP > 50 mmHg n (%)	7 (1.6)
Isolated LV dysfunction n (%)	20 (4.7)
Isolated RV dysfunction n (%)	6 (1.4)
Biventricular dysfunction n (%)	4 (0.9)
Pericardial effusion n (%)	40 (9.4)

ARDS: acute respiratory distress syndrome, CK-MB: Creatine Kinase MB, NT-proBNP: N-terminal pro-b-type natriuretic peptide, CRP: C-reactive protein, WBC: blood cell, AST: aspartate transferase, ALT: Alanine Aminotransferase, LBBB: left bundle branch block, RBBB: right bundle branch block, EDD: end-diastolic dimension, ESD: end-systolic dimension, EF: ejection fraction, FS: fractional shortening, RVSP: right ventricular systolic pressure.

**Table 2:** Outcome measures.

Outcomes	Number of Patients (%)
In-hospital-all-cause-mortality	12 (2.8)
Biventricular dysfunction	4 (0.9)
Probable myocarditis	20 (4.7)
Shock	34 (8.0)
AF	10 (2.3)
CHF	1 (0.2)
MODS	56 (13.1)
AKI	78 (18.3)
Need for hemodialysis	22 (5.2)

AF: atrial fibrillation, CHF: congestive heart failure, MODS: multi-organ dysfunction, AKI: acute kidney injury.

**Table 3:** Multivariable regression analysis for the prediction of mortality and overall complications.

Multivariable Regression Analysis in the Prediction of Mortality				Multivariable Regression Analysis in the Prediction of Overall Complications (Mortality/Probable Myocarditis/CHF/MODS/Need for Hemodialysis/AKI))			
Variable	Exp (B)	95% C.I for Exp (B)	*p* Value	Variable	Exp (B)	95% C.I for Exp (B)	*p* Value
Age	1.02	0.97–1.081	0.279	Age	1.02	1.009–1.046	0.004
Gender (Female)	2.85	0.62–12.96	0.174	Gender (female)	0.48	0.303–0.789	0.003
Hypertension	0.97	0.19–4.93	0.971	Hypertension	1.13	0.622–2.08	0.676
Diabetes	1.77	0.43–7.28	0.423	Diabetes	1.53	0.901–2.61	0.115
Eschar	1.30	0.25–6.61	0.745	Eschar	1.46	0.755–2.85	0.257
Probable myocarditis	9.79	1.88–50.79	0.007	Tachycardia	1.36	0.727–2.57	0.332
MODS	7.45	1.65–33.66	0.009	ARDS	1.47	0.802–2.72	0.210
Need for hemodialysis	3.35	0.42–26.64	0.252	LV systolic dysfunction	22.99	6.383–82.86	<0.001
AKI	1.56	0.26–9.41	0.624				

CHF: congestive Heart failure, MODS: multi-organ dysfunction, AKI: acute kidney injury, ARDS: acute respiratory distress syndrome, LV: left ventricle.

## Data Availability

The data presented in this study are available from the corresponding author upon reasonable request, due to ethical restrictions on data sharing.
